# The Significance of the *CLDN18-ARHGAP* Fusion Gene in Gastric Cancer: A Systematic Review and Meta-Analysis

**DOI:** 10.3389/fonc.2020.01214

**Published:** 2020-09-02

**Authors:** Wei-Han Zhang, Shou-Yue Zhang, Qian-Qian Hou, Yun Qin, Xin-Zu Chen, Zong-Guang Zhou, Yang Shu, Heng Xu, Jian-Kun Hu

**Affiliations:** ^1^State Key Laboratory of Biotherapy, Department of Gastrointestinal Surgery and Laboratory of Gastric Cancer, West China Hospital, Sichuan University and Collaborative Innovation Center of Biotherapy, Chengdu, China; ^2^State Key Laboratory of Biotherapy, West China Hospital, Sichuan University and Collaborative Innovation Center, Chengdu, China; ^3^Department of Radiology, West China Hospital, Sichuan University, Chengdu, China; ^4^State Key Laboratory of Biotherapy, West China Hospital, Department of Gastrointestinal Surgery and Laboratory of Digestive Surgery, Sichuan University, and Collaborative Innovation Center for Biotherapy, Chengdu, China

**Keywords:** gastric cancer, fusion gene, *CLDN18-ARHGAP*, survival, therapy

## Abstract

**Objective:** The objective of this study was to summarize the clinicopathological characteristics of the *CLDN18-ARHGAP* fusion gene in gastric cancer patients.

**Background:** The *CLDN18-ARHGAP26* fusion gene is one of the most frequent somatic genomic rearrangements in gastric cancer, especially in the genomically stable (GS) subtype. However, the clinical and prognostic meaning of the *CLDN18-ARHGAP* fusion in gastric cancer patients is unclear.

**Methods:** Studies that investigated *CLDN18-ARHGAP* fusion gastric cancer patients were identified systematically from the PubMed, Cochrane, and Embase databases through the 28th of February 2020. A systematic review and meta-analysis were performed to estimate the clinical significance of *CLDN18-ARHGAP* fusion in patients.

**Results:** A total of five eligible studies covering 1908 patients were selected for inclusion in the meta-analysis based on specified inclusion and exclusion criteria. Several fusion patterns were observed linking *CLDN18* and *ARHGAP26* or *ARHGAP6*, with the most common type being *CLDN18/exon5*-*ARHGAP26/exon12*. The survival outcome meta-analysis of the *CLDN18-ARHGAP* fusion gene showed that it was associated with overall survival outcomes in gastric cancer (HR, 2.03, 95% CI 1.26–3.26, *P* < 0.01, random-effects). In addition, diffuse gastric cancer had a greater proportion of CLDN18-ARHGAP fusions than intestinal gastric cancer (13.3%, 151/1,138 vs. 1.8%, 8/442; *p* < 0.001). Moreover, gastric cancer patients with the *CLDN18-ARHGAP* fusion gene are more likely to be female or have a younger age, lymph node metastasis and advanced TNM stages.

**Conclusion:** The *CLDN18-ARHGAP* fusion is one of the molecular characteristics of diffuse gastric cancer and is also an independent prognostic risk factor for gastric cancer. In addition, it is also related to multiple clinical characteristics, including age, sex, lymph node metastasis and tumor stage. However, the mechanism of the *CLDN18-ARHGAP* fusion gene and potential targeted therapeutic strategies need further exploration.

## Introduction

Recurrent chromosomal translocations have been implicated in multiple tumor types. It has been demonstrated that frequent fusion genes are involved in oncogenesis and progression as driver events. The Philadelphia chromosome is well reported as the first cancer-associated chromosomal rearrangement, resulting in the *BCR-ABL* fusion, which was also identified as a diagnostic feature and therapeutic target of chronic myeloid leukemia patients. Thereafter, oncologic fusion genes were frequently identified in leukemia, lymphoma and sarcoma, but with a relatively low incidence rate in epithelial tumors (1). For epithelial tumors, the most well-known fusion gene is the *EML4-ALK* gene, which was detected in ~5–10% of non-small cell lung cancer patients (2, 3).

Regarding gastric cancer, The Cancer Genome Atlas (TCGA) project proposes the molecular classification of gastric cancers and divides them into four separate subtypes, and the *CLDN18-ARHGAP26* fusion gene is highly enriched in the genomically stable (GS) subtype (4). Histologically, the majority of GS subtype cancers were the diffuse type according to the Lauren classification, with common somatic mutations located in the *CDH1* and *RHOA* genes. It is notable that *CLDN18-ARHGAP* fusions were mutually exclusive with *RHOA* mutations in the classification of TCGA (4). A subsequent functional study indicated that the introduction of the *CLDN18-ARHGAP26* fusion to tumor cells can direct the loss of the epithelial phenotype, epithelial-mesenchymal transition, and inhibition of the RHOA signaling pathway and contribute to tumor invasiveness in cancer cell lines (5). Our study group previously used a whole-genome sequencing approach to characterize the genomic features of signet ring cell gastric cancer and identified frequent *CLDN18-ARHGAP* fusions (6). More importantly, we linked multiple clinical characteristics with *CLDN18-ARHGAP28/6* fusions, including the proportion of signet ring cell content, TNM stage, and poor prognosis with the current chemotherapy strategy (6). These findings were quickly validated by an independent Japanese study (7, 8). Meanwhile, a further Korean study found that the *CLDN18-ARHGAP* fusion gene can promote the invasion and migration capacity of gastric cancer cells (9). However, there is a lack of studies systematically evaluating the clinicopathological characteristics and prognostic meaning of *CLDN18-ARHGAP* fusions in gastric cancers.

Therefore, in this meta-analysis and systematic review, we will systematically summarize and assess the clinical significance and advances of the *CLDN18-ARHGAP* fusion gene in gastric cancer. The primary endpoint of the present study is the survival outcomes of patients with the *CLDN18-ARHGAP* fusion gene, and other endpoints are the relationship of the *CLDN18-ARHGAP* fusion gene with tumor-related clinicopathological characteristics, such as age, sex, tumor location and tumor stage.

## Methods

### Search Strategy

We searched the Web of Knowledge, PubMed, Embase and Cochrane Collaborative Center Register of Controlled Trials databases on the 28th of February 2020 by using the terms “gastric cancer,” “gastric carcinoma,” “gastric neoplasm,” “stomach cancer,” “stomach carcinoma,” “stomach neoplasm,” “CLDN,” “claudin,” “ARHGAP,” “Rho GTPase-activating protein,” “oligophrenin-1-like” and “OPHN1L” and strictly restricted search results to titles, abstracts and keywords. We also searched previously published meta-analyses and systematic reviews. All of those articles were independently screened by two authors (WH Zhang and SYZ) based on the inclusion and exclusion criteria of the study. Because the studies included in this meta-analysis have been published, ethical approval was not needed from ethics committees. The results of this study were reported according to the PRISMA statement (10).

### Study Selection

Those studies that reported the relationship between the *CLDN18-ARHGAP* fusion gene and the clinicopathological characteristics or survival outcomes of gastric cancer patients were included. The exclusion criteria included the following: (1) mixed benign disease of the stomach; (2) articles in languages other than English; and (3) incomplete data or duplicated data. For studies with more than one article and with duplicated data, only the article with the most complete data was included for analysis in this study.

### Data Extraction and Quality Assessment

Data from the included studies were independently extracted by two authors (WH Zhang and QQ Hou). For each study, we recorded the following information: name of the first author, year of publication, country of the study, study design, time period of the study and examined method for the *CLDN18-ARHGAP* fusion gene. Furthermore, the following clinicopathological characteristics were also extracted and included in the present study: fusion types of the *CLDN18* and *ARHGAP* genes, age (years), sex (male or female), tumor location (upper third of stomach), tumor stage (T stage, N stage and TNM stage) and survival outcomes between *CLDN18-ARHGAP* fusion-positive and CLDN18-ARHGAP fusion-negative patients. The patients were divided into a *CLDN18-ARHGAP* fusion-positive group and a *CLDN18-ARHGAP* fusion-negative group according to the status of the expression of *CLDN18-ARHGAP26*/6 fusions.

The quality assessment of the included studies was evaluated by two authors (WH Zhang and QQ Hou) independently. Retrospective studies were assessed by the Newcastle–Ottawa Scale (NOS), which is a 9-point scale (11). Studies with NOS scores lower than 6 were deemed moderate or low-quality studies. Any disagreements regarding the quality assessment were resolved by discussion with supervisors (H Xu and JK Hu).

### Statistical Analysis

This study was performed according to the Cochrane guidelines (12). For studies that only reported the medians and ranges for continuous variables, the data were converted to means and standard deviations (SDs) with the method reported by Hozo et al. (13). Categorical variables are presented as ratios and were analyzed by the Mantel-Haenszel method, and continuous variables are presented as the mean ± SD and were analyzed by the inverse variance method. The odds ratio (OR) and mean difference (MD) were used to evaluate dichotomous and continuous data, respectively. The hazard ratio (HR) was used to evaluate survival outcomes. The OR, HR and MD were reported with 95% confidence intervals (CIs). Heterogeneity among studies was assessed by the I^2^ value. According to the I^2^ value, the studies were determined to have low (I^2^ < 30%), moderate (30–50%) or considerable (I^2^ ≥ 50%) heterogeneity. Begg's test was used to assess publication bias. For the survival analysis, we updated the survival information to Jan 2019 of our previous study (all 829 patients, Shu et al.) (6). In addition, individual survival information from the TCGA cohort was also updated according to a recent report from the TCGA research network (14). Survival information from other studies was extracted with the method reported by Tierney et al. by Engauge Digitizer software (Version 11.2) (15). A *P*-value < 0.05 was considered statistically significant for the present study. All of the statistical analyses were performed by R software (http://www.R-project.org/) with the “survival,” “survminer,” “ggplot2,” “meta,” and “metafor” packages.

## Results

### General Characteristics

**Figure 1:**
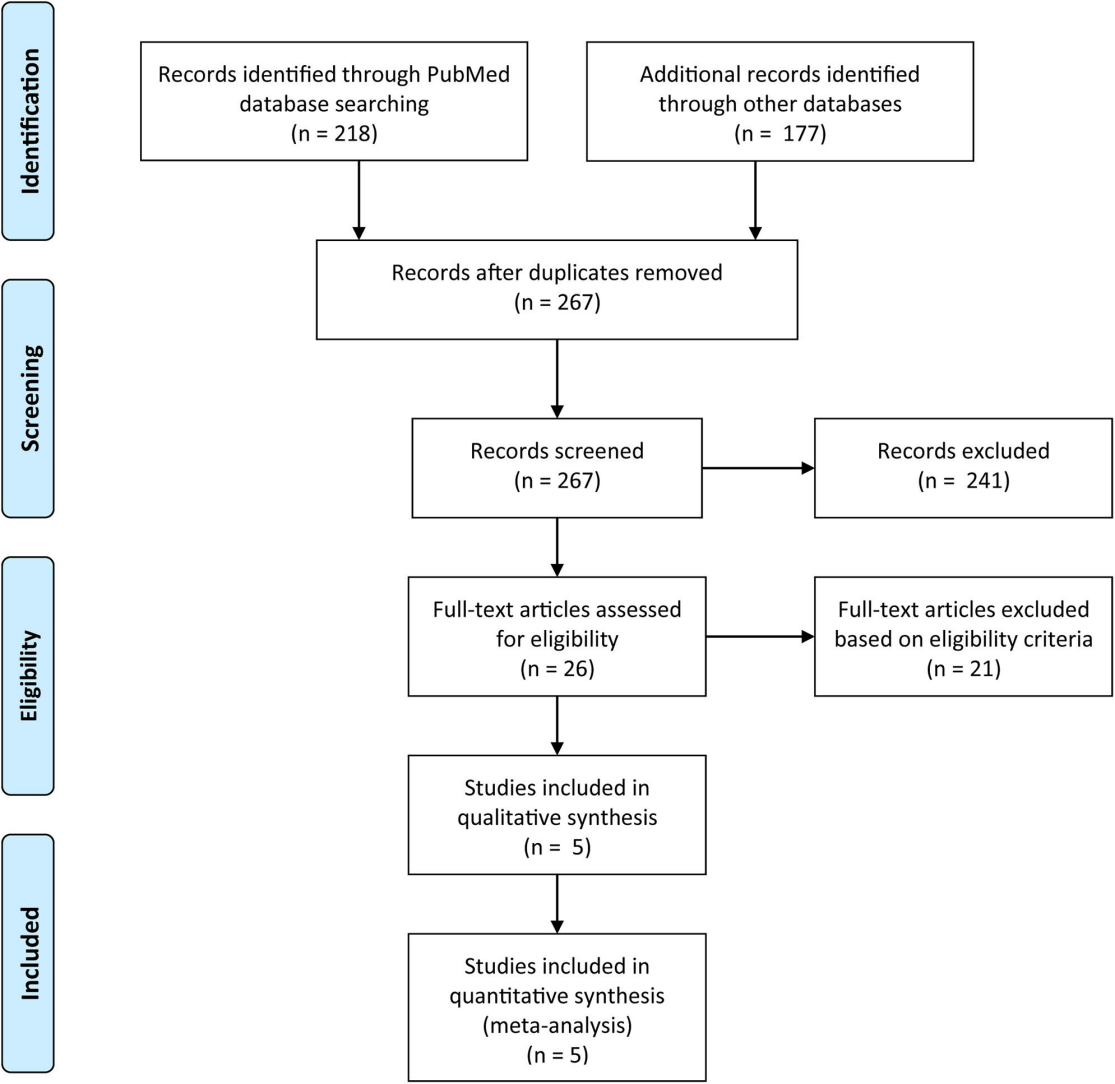
PRISMA flow chart.

We retrieved 395 records with 128 duplicates. After reading the titles and abstracts, 26 articles remained for reassessment according to their full texts. After reading the full texts of these articles, we included five studies that presented the relationship of clinicopathological characteristics and survival outcomes with *CLDN18-ARHGAP* fusion gene status (Figure 1, PRISMA Flow Diagram). We also evaluated the quality of all included studies with the Newcastle-Ottawa Scale, and the results showed that all studies had a score ≥ 6.

All 5 studies were from four countries (United States, Japan, Korea and China) and were published between 2014 and 2019. Finally, a total of 1,908 patients were included in the present study: 151 (7.9%) patients in the *CLDN18-ARHGAP* fusion-positive group and 1,757 (92.1%) patients in the *CLDN18-ARHGAP* fusion-negative group. General characteristics of those included five studies were summarized in Table 1. There were several fusion types in *CLDN18-ARHGAP* fusion gene gastric cancer patients. Also, the fusion types from the five reported studies between *CLDN18 and ARHGAP* genes are presented in Table 2.

**Table 1:** Characteristics of studies reported clinicopathological characteristics between *CLDN18-ARHGAP* fusion and gastric cancers.

**Study**	**Country**	**Period**	**Samples**	**Lauren Type**			***CLDN18-ARHGAP*** ***Fusion positive***			**Tumor stage**	**Examine methods**	**NOS^**#**^**	**Special** **Characteristics**
				***N*** **=** **(%)**			***N*** **=** **(%)**						
				DGC^*^	**IGC**	**NA**	**All**	**DGC**	**IGC**				
Nakayama et al. (7)	Japan	2006–2015	146	136 (93.2)	10 (6.8)	NA	22 (15.1)	22 (16.2)	0 (0.0)	I–IV	Fusion-FISH, RNA-seq	7	Young age
													Patients (≤ 40)
Shu et al. (6)	China	2009–2014	829	358 (43.2)	154 (18.6)	317 (38.2)	73 (8.8)	55 (15.4)	2 (1.3)	I–IV	WGS, RT-PCR	8	Fusion related to the proportion of SRCC
Tanake et al. (8)	Japan	2000–2013	254	172 (67.7)	82 (32.3)	0	26 (10.2)	22 (12.8)	4 (4.9)	I–IV	RT-PCR, FISH	6	Fusion-positive DGCs E-cad expression
Yang et al. (9)	Korea	2003–2017	384	384 (100.0)	0	0	17 (4.4)	17 (4.4)	0	I–IV	RT-PCR, RNA seq	6	Fusion related to H. pylori infections
TCGA (4)	United State	NM	295	88 (29.8)	196 (66.4)	11 (3.7)	13 (4.4)	10 (11.4)	2 (1.0)	I–IV	WGS or RNA-seq	8	Fusion related to GS tumors

* *Included mixed type*.

# *NOS, The Newcastle-Ottwa Scale (11)*.

*DGC, diffuse gastric cancer; IGC, intestinal gastric cancer; TCGA, The Cancer Genome Atlas; WGS, whole-genome sequence; RNA-seq, RNA sequence; RT-PCR, reverse transcription-polymerase chain reaction; FISH, fluorescence in situ hybridization, SRCC, signet ring cell cancer; GS, genomically stable; NA, not applicable; NM, not mentioned*.

**Table 2:** The *CLDN18-ARHGAP* fusion models in reported studies.

**Study**	**Number of** ***CLDN18-ARHGAP* fusion**	**Fusion Mode**		**Numbers** **(%)**
Nakayama et al. (7)	22	*CLDN18/exon5*	*ARHGAP26/exon10*	18 (81.8)
			*ARHGAP26/exon 12*	
		*CLDN18/exon5*	*ARHGAP6/exon2*	2 (9.1)
		*CLDN18/exon5*	*ARHGAP42/exon7*	1 (4.5)
		*CLDN18/exon5*	*ARHGAP10/exon8*	1 (4.5)
Shu et al. (6)	73	*CLDN18/exon5*	*ARHGAP26/exon12*	58 (79.5)
		*CLDN18/exon5*	*ARHGAP26/exon10*	7 (9.6)
		*CLDN18/exon4*	*ARHGAP26/exon11*	1 (1.4)
		*CLDN18/exon5*	*ARHGAP6/exon2*	7 (9.6)
Tanake et al. (8)	26	*CLDN18/exon5*	*ARHGAP26/exon12*	24 (92.3)
		*CLDN18/exon5*	*ARHGAP26/exon10*	1 (3.8)
		*CLDN18/exon5*	*ARHGAP6/exon2*	1 (3.8)
Yang et al. (9)	17	*CLDN18*	*ARHGAP26*	13 (76.5)
		*CTNND1*	ARHGAP26^*^	2 (11.8)
		*ANXA2*	MYO9A^*^	2 (11.8)
TCGA (4)	13	*CLDN18/exon5*	*ARHGAP26/exon12*	10 (76.9)
		*CLDN18/exon5*	*ARHGAP26/exon10*	1 (7.7)
		*CLDN18/exon5*	*ARHGAP6/exon2*	2 (15.4)

*TCGA, The Cancer Genome Atlas*.

* *Also known as RhoGAP domain-containing fusions*.

The CLDN18/exon5-ARHGAP26/exon12 fusion was the most common fusion pattern among present reports and is clearly understood. In addition, the mutation counts between the CLDN18-ARHGAP fusion-positive and CLDN18-ARHGAP fusion-negative patients in cohorts of Shu et al. and TCGA were analyzed (Supplementary Tables 1, 2). However, there was no overlap of the significant mutation counts gene between CLDN18-ARHGAP fusion-positive and -negative patients in the Shu and TCGA cohorts. Different pathological subtypes and the sample size difference of the two cohorts may be the reasons for the results. However, the study of Shu et al. was based on the whole genome sequencing, so we cannot analyze the RNA expression level between fusion positive and negative patients. Therefore, we analyzed copy number variation in the cohort of Shu et al. (Supplementary Table 3) and the RNA expression level (Supplementary Table 4) in the TCGA cohort between CLDN18-ARHGAP fusion-positive and CLDN18-ARHGAP fusion-negative gastric cancer patients.

In the meta-analysis of clinicopathological characteristics, the *CLDN18-ARHGAP* fusion gene-positive group had more patients with a younger age (MD: −5.85, 95% CI: −11.22 to −0.48, *p* = 0.03), a lower proportion of male patients (OR: 0.40, 95% CI 0.23–0.70, *p* = 0.001), patients with a more advanced N stage (OR: 3.41, 95% CI 2.00–5.82, *p* < 0.001) and patients with a more advanced TNM stage (OR: 3.07, 95% CI 1.56–6.05, *p* = 0.001) than the *CLDN18-ARHGAP* fusion gene-negative group (Table 3). However, tumor location (*p* = 0.43) and T stage (*p* = 0.07) were not significantly different between the two groups. Moreover, we found that diffuse gastric cancers had a greater proportion of *CLDN18-ARHGAP* fusion genes than intestinal gastric cancers (13.3%, 151/1,138 vs. 1.8%, 8/442; *p* < 0.001).

**Table 3:** The Meta-analysis of clinicopathological characteristics between patients with *CLDN18-ARHGAP* fusion positive and negative patients.

**Characteristics**	**Included Study**	***CLDN18-ARHGAP*** **Fusion (+)** ***N* = (%)**	***CLDN18-ARHGAP*** **fusion (–)** ***N*= (%)**	**Test of Heterogeneity**			**Meta-analysis**		
				**χ^2^**	***I*^**2**^ (%)**	***p*-value**	**OR or MD**	**95% CI**	***p*-value**
Age (years)	(4, 6, 8)	112	1177	8.76	77	0.01	−5.85	−11.22 to −0.48	0.03
Gender (Male)	(4, 6–9)	61/151 (40.4)	1,107/1,668 (66.4)	8.51	53	0.07	0.40	0.23–0.70	0.001
Tumor Location (Upper)	(4, 6–9)	31/151 (20.5)	621/1,668 (37.2)	14.19	72	0.007	0.68	0.27–1.75	0.43
T stage (T2–T4)	(4, 6–8)	116/134 (86.6)	1,131/1,301 (86.9)	0.63	0	0.89	1.76	0.96–3.22	0.07
*N* stage (*N*+)	(4, 6–8)	110/134 (82.1)	902/1,301 (69.3)	3.33	10	0.34	3.41	2.00–5.82	<0.001
TNM stage (III–IV)	(4, 6, 7, 9)	120/151 (79.5)	1,046/1,668 (62.7)	7.66	48	0.10	3.07	1.56–6.05	<0.001

*95% CI, 95% confidence interval; MD, mean difference; OR, odds ratio*.

### Survival Analysis

**Figure 2:**
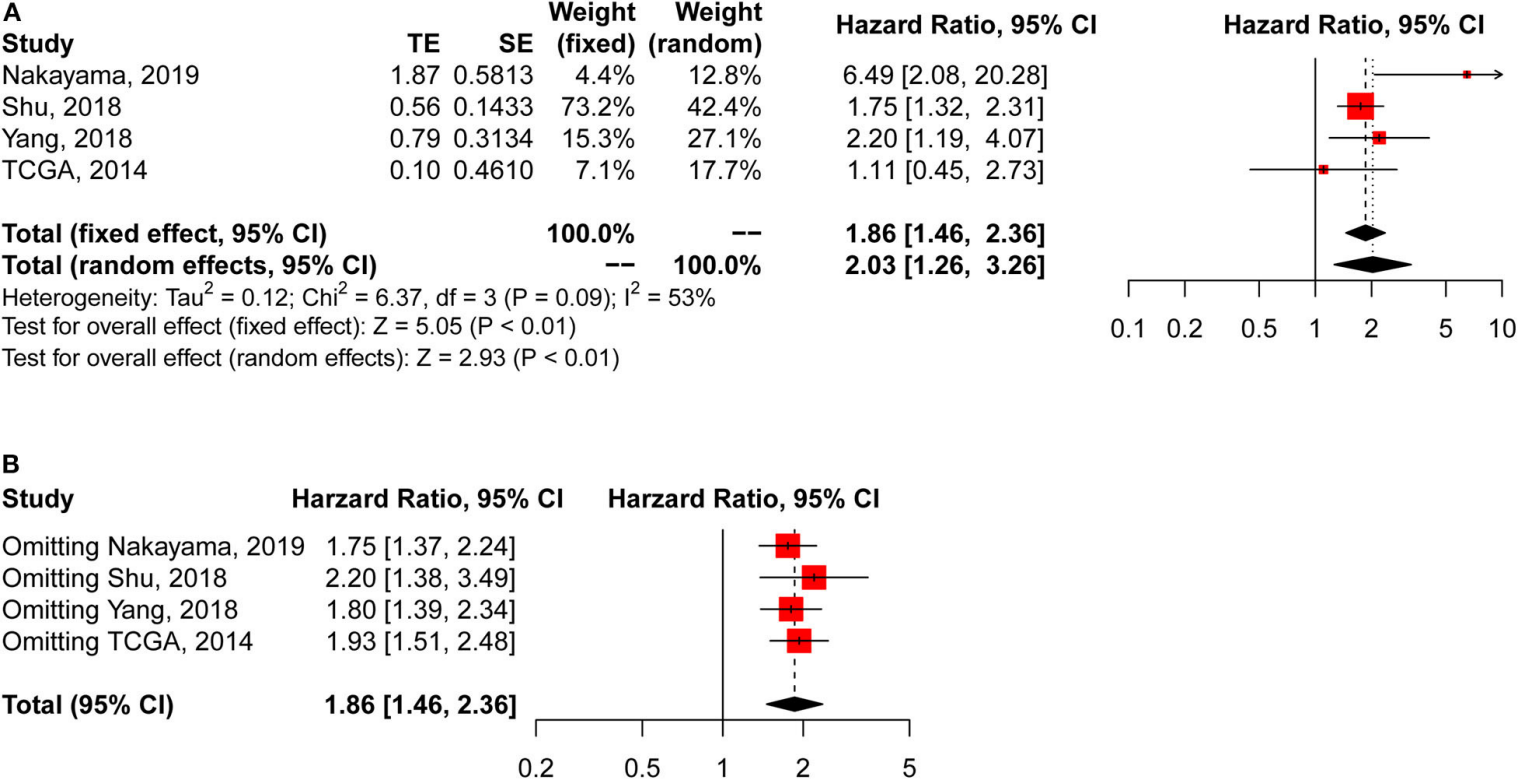
Meta-analysis of survival outcomes between CLDN18-ARHGAP fusion-negative and CLDN18-ARHGAP fusion-positive gastric cancer patients. **(A)** Survival outcomes of the CLDN18-ARHGAP26 fusion gene (positive vs. negative); **(B)** sensitivity analysis of the included studies.

*CLDN18-ARHGAP* fusion-positive gastric cancer patients had significantly poorer overall survival outcomes than *CLDN18-ARHGAP* fusion-negative patients in the meta-analysis (HR: 2.03, 95% CI 1.26–3.26, *p* < 0.01, random effects) (Figure 2A), and the survival results in the meta-analysis were relatively stable in the sensitivity analysis (Figure 2B). Because there were only 4 studies included in the survival analysis, publication bias was only evaluated by Begg's test. The results demonstrated that there was no publication bias according to Begg's test with continuity correction (*p* = 0.555).

**Figure 3:**
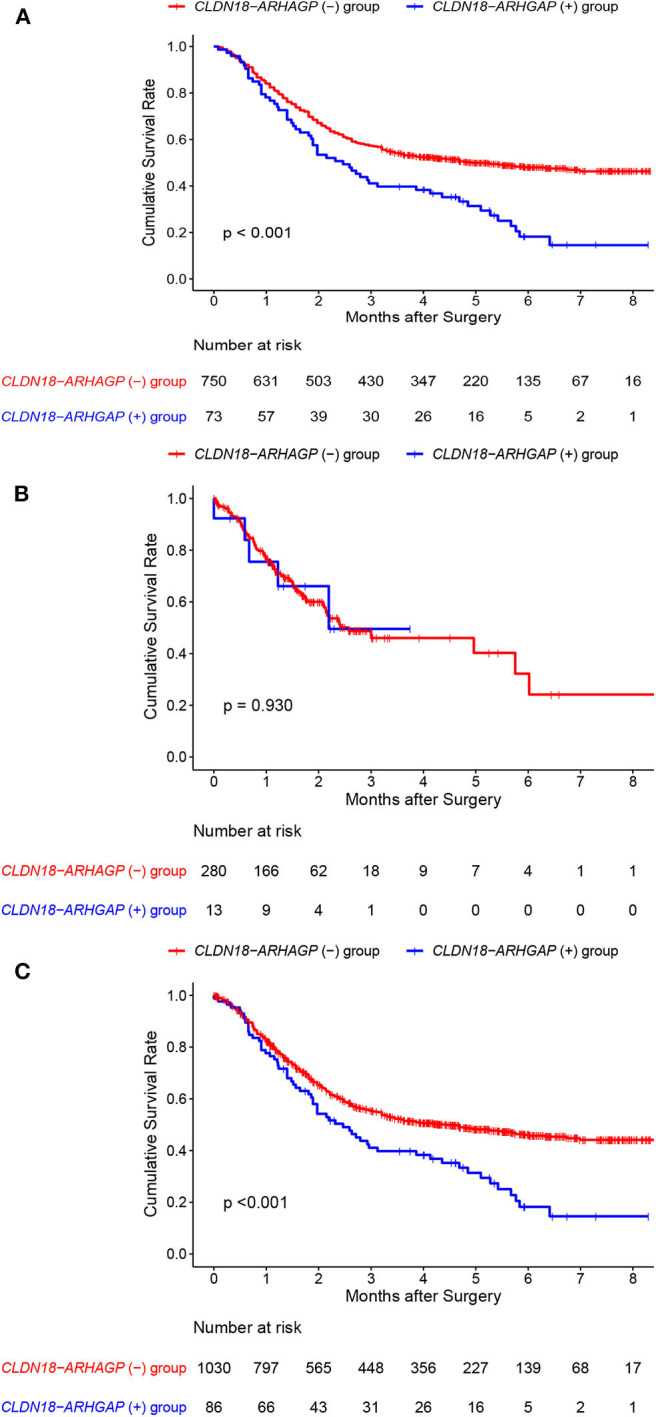
Individual overall survival outcomes of CLDN18-ARHGAP26/6 fusion gastric cancer patients in the Shu et al. and TCGA cohorts. **(A)** Overall survival outcomes in the Shu et al. cohort; **(B)** overall survival outcomes in the TCGA cohort; **(C)** overall survival outcomes in the two cohorts.

In addition, we acquired updated individual survival information from the cohort of Shu et al. and the TCGA cohort. Therefore, the survival difference between *CLDN18-ARHGAP* fusion-positive and *CLDN18-ARHGAP* fusion-negative patients was evaluated in these two cohorts (Figures 3A,B). A significant survival difference was found between the *CLDN-ARHGAP* fusion gene-positive and *CLDN-ARHGAP* fusion gene-negative groups with the combination of the data from the two cohorts (*p* < 0.001) (Figure 3C). In addition, the Cox proportional hazards model was used to present the independent prognostic risk factors in the merged data of the Shu and TCGA cohorts (Table 4). In multivariate survival analysis, positive *CLDN18-ARHGAP* fusion (HR: 1.365, 95% CI 1.031–1.809, *p* = 0.030) and TNM stage (stage III vs. stage I, HR: 3.018, 95% CI 1.763–5.164, *p* < 0.001; stage IV vs. stage I, HR: 7.155, 95% CI 4.083–12.538, *p* < 0.001) were independent prognostic risk factors.

**Table 4:** Univariate and Multivariate survival analysis of *CLDN18-ARHGAP26/6* fusion gene in TCGA and Shu cohort.

**Characteristics**		**Univariate Analysis**			**Multivariate Analysis**		
		**OR**	**95% CI**	***P*-value**	**OR**	**95% CI**	***P*-value**
Age (years)	<65 vs. ≥65	1.14	0.877–0.959	0.136	1.343	1.125–1.603	0.090
Gender	Male vs. Female	1.042	0.868–1.252	0.658	0.988	0.817–1.195	0.900
Tumor location	Upper vs. Other	0.973	0.810–1.168	0.769	0.868	0.722–1.044	0.134
T stage	T2 vs. T1	3.119	0.960–10.130	0.058			
	T3 vs. T1	3.953	1.253–12.470	0.019			
	T4 vs. T1	6.455	2.073–20.100	0.001			
N stage	N1 vs. N0	1.999	1.379–2.897	<0.001			
	N2 vs. N0	2.453	1.733–3.471	<0.001			
	N3 vs. N0	3.94	2.888–5.376	<0.001			
TNM stage	II vs. I	1.29	0.725–2.294	0.386	1.339	0.753–2.385	0.32
	III vs. I	2.864	1.668–4.889	<0.001	3.018	1.763–5.164	<0.001
	IV vs. I	6.669	3.825–11.628	<0.001	7.155	4.083–12.538	<0.001
*CLDN18-ARHGAP26/6* fusion	Positive vs. Negative	1.629	1.247–2.127	<0.001	1.365	1.031–1.809	0.03

*OR, odds ratio; 95% CI, 95% confidence interval*.

* *Only TNM stage entered into the Cox regression model due to the potentially confounding effect*.

## Discussion

In the present study, a total of 5 cohort studies reported the presence of *CLDN18-ARHGAP* fusions and its relationship with clinicopathological characteristics and survival outcomes in gastric cancer patients. Multiple clinical characteristics were observed to be correlated with the frequency of *CLDN18-ARHGAP* fusions. Finally, significant enrichment of *CLDN18-ARHGAP* fusions was observed in female patients and patients with a younger age, diffuse gastric cancer by Lauren classification and more advanced tumor stages (N stage and TNM stage), but *CLDN18-ARHGAP* fusions were not related to the primary tumor location. Most importantly, the *CLDN18-ARHGAP* fusion was significantly related to poor survival outcomes in the meta-analysis (HR: 2.03, 95% CI 1.26–3.26, *p* < 0.01, random effects). Meanwhile, in the survival analysis with the combination of individual data from the Shu et al. and TCGA cohorts, the CLDN18-ARHGAP fusion was an independent prognostic risk factor for overall survival outcomes (HR: 1.365, 95% CI 1.031–1.809, *p* = 0.030).

The *CLDN18-ARHGAP* fusion gene is formed by chromosomal rearrangements of the *CLDN18* and *ARHGAP* genes, mainly *CLDN18-ARHGAP26* and *CLDN18-ARHGAP6* fusions. *CLDN18-ARHGAP26*/6 contains a nearly full coding region of *CLDN18* and the conserved domain of *ARHGAP26*/6. Functionally, the *CLDN18* gene encodes the claudin-18 protein, which forms tight junctions in epithelial cells. The CLDN18-ARHGAP fusion protein may disrupt the structure of the wild-type CLDN18 protein, which may impact the cellular adhesion of cancer cells. The *ARHGAP26* gene encodes the ARHGAP26 protein, a member of the Rho GTPase activating the protein, which is also known as the GTPase regulator associated with focal adhesion kinase (GRAF). GRAF not only regulates the activity of RHOGAP family proteins (16) but also coordinates membrane remodeling, which is necessary for the CLIC/GEEC endocytic pathway (17). Regev et al. suggested that the GRAF protein may play a role in the maintenance of the normal epithelial phenotype, the depletion of which can induce a neoplastic transformation-related epithelial-mesenchymal transition (EMT)-like process (18). For the fusion proteins of CLDN18-ARHGAP, the large segment of ARHGAP fuses to the carboxy terminus of CLDN18 and retains the carboxy-terminal GAP domain, which may affect ARHGAP's regulation of the RHOA pathway and/or the epithelial phenotype of gastric cancer cells. Several studies have indicated that the introduction of the *CLDN18-ARHGAP26* fusion in cancer cells can increase their migration and invasion ability (5, 6), which can partially explain the advanced tumor stages in *CLDN18-ARHGAP* fusion-positive patients.

In the TCGA gastric cancer cohort, the *CLDN18-ARHGAP26/6* fusion was enriched in patients with the genomically stable subtype, which has higher frequency of lower third tumors, patients with a younger age and more diffuse histological subtype tumors (4). In our previous study, we found that the *CLDN18-ARHGAP26/6* fusion was significantly associated with the proportion of signet ring cancer cells and tumor stage (6). Tanaka et al. also observed a higher frequency of *CLDN18-ARHGAP26/6* fusions in diffuse gastric cancers than in intestinal gastric cancers (22/172, 12.8% vs. 4/82, 4.8%) (8). In the present study, we found a significant difference in the frequency of the *CLDN18-ARHGA*P fusion gene between diffuse gastric cancer and intestinal gastric cancer (13.3%, 151/1,138 vs. 1.8%, 8/442; *p* < 0.001). Therefore, the *CLDN18-ARHGAP* fusion may be an important molecular characteristic of diffuse gastric cancer.

It is well reported that the diffuse subtype has a significantly poorer prognosis than the intestinal subtype of gastric cancer according to the Lauren classification, probably because of the more advanced tumor stages and potential resistance to traditional chemotherapy regimens of diffuse gastric cancers (19). We previously reported the prognostic value of the *CLDN18-ARHGAP26/6* fusion, which is a risk factor for overall survival and confers postoperative chemotherapy resistance (6). The present study summarized the survival outcomes of previously reported studies focused on the C*LDN18-ARHGAP* fusion gene. Thereafter, the survival outcome meta-analysis showed that patients with *CLDN18-ARHGAP* fusion have a significantly poorer prognosis than patients without *CLDN18-ARHGAP* fusion. Due to the enrichment of the *CLDN18-ARHGAP* fusion in patients with more advanced stages, it is important to assess the factors independently associated with the *CLDN18-ARHGAP* fusion. Multivariate analyses of individual data from the Shu and TCGA cohorts presented a significant association of the *CLDN18-ARHGAP* fusion status with poor treatment outcomes after adjusting for tumor stage, indicating that the *CLDN18-ARHGAP* fusion is an independent prognostic factor for gastric cancers. In addition, it is necessary to mention that some of the included studies were not traditional clinical studies, and limited follow-up durations (such as that in the TCGA cohort) may increase the bias risk in the survival analysis above.

According to a previous study (6), gastric cancer patients with the *CLDN18-ARHGAP26/6* fusion gene cannot obtain survival benefits from 5-FU/oxaliplatin-based chemotherapy, which may partially explain the poor prognosis of *CLDN18-ARHGAP* fusion patients. However, no other study analyzed the relationship between the *CLDN18-ARHGAP26* fusion gene and chemotherapy drug therapeutic sensitivity. Mechanistically, resistance to these chemotherapy drugs was observed after the introduction of the *CLDN18-ARHGAP26/6* fusion into cell lines (6). Because no reported gastric cancer cell lines carry *CLDN18-ARHGAP26/6* fusions according to the Cancer Cell Line Encyclopedia database (20, 21), patient-derived xenograft (PDX) and organoid models may be the breakthrough point for future research to help validate drug resistance, screen fusion-targeted drugs, and guide personalized therapy (22–24). Yan et al. described a gastric cancer organoid model that can be used to assess the efficacy of chemotherapy (25). Unfortunately, no patient with *CLDN18-ARHGAP26* fusion was captured in their gastric cancer organoid bank. Nakayama et al. established two *CLDN18-ARHGAP26* fusion-positive cell lines from 125 gastric cancer PDXs (26). Collectively, these results suggest that the establishment of PDX and organoid models can help researchers conduct drug sensitivity screening and explore personalized medicine applications for therapy response testing in the future.

Specifically, the aberrant activation of claudin-18 splice variant 2 (claudin-18.2) was detected in multiple types of cancer compared with its limited expression in normal tissues. The rate of claudin-18.2-positive patients was more than 80% according to a Japanese study on gastric cancer, and more than 40% of patients had moderate-to-strong expression (≥ 2+ membrane staining intensity in ≥ 40% of tumor cells) in both the primary tumor and metastatic lymph nodes (27). Claudin-18.2 has been considered a novel druggable target for some epithelial tumors (28). Indeed, a chimeric monoclonal antibody drug has been recently developed (i.e., zolbetuximab, formerly known as IMAB362), which induces the immune-mediated lysis of CLDN18.2-positive cancer cells by activating immune effector mechanisms (29). Clinical trials evaluating the safety and efficacy of zolbetuximab for claudin-18.2-positive cancer patients are ongoing. The up-to-date evidence is promising but remains to be validated by high-quality clinical trials (30, 31). We also noticed that there is an ongoing clinical trial, which is focused on safety and efficacy of anti-claudin18.2 chimeric antigen receptor t-cell (CAR-T) immunotherapy in patients with advanced gastric cancer or pancreatic cancer (ClinicalTrials.gov Identifier: NCT03159819). The final results of the anti-claudin-18.2 CAR-T immunotherapy as a new anti-tumor targeted immunotherapy study are highly anticipated. Considering that a higher claudin-18.2 positive rate was observed in patients with the diffuse subtype of gastric cancer than in those with the intestinal type (57.5 vs. 39.0%) (27), an unsolved and important question is whether the *CLDN18-ARHGAP* fusion gene is correlated with claudin-18.2 protein expression and thus is suitable for zolbetuximab treatment, which requires solid clinical evidence.

According to previous studies, whole-genome sequencing, RNA sequencing, reverse transcription-polymerase chain reaction (RT-PCR) and fluorescence *in situ* hybridization (FISH) are all effective methods for the detection of *CLDN18-ARHGAP* fusions. Once the clinical significance of the *CLDN18-ARHGAP* fusion has been proven and potential target treatment regimens have been determined, establishing a stable and effective detection method for this fusion gene is particularly important. Considering the preservation of tumor tissue as well as the stability and economic cost of the examination, FISH may be the first choice for promotion in clinical practice. In addition, RNA *in situ* hybridization techniques may be useful in the detection of fusion genes. However, the sensitivity and specificity of the detection of fusion genes by such methods should be validated by clinical studies. In addition, oncogenic fusion circRNAs (f-circRNAs) derived from cancer-associated chromosomal translocations exhibit properties of tumor-promoting cellular transformation, cell viability and resistance to treatment (32, 33). Consistently, f-circRNAs derived from *SLC32A2-ROS1* and *EML4-ALK* fusion genes, which have been determined as biomarkers for the use of targeted drugs in lung cancer, were also demonstrated to impact cell migration, invasion and cell proliferation in lung cancer cells (34, 35). More importantly, the f-circRNAs of *EML4-ALK* can be detected in the plasma of *EML4-ALK*-positive NSCLC patients (36). These results suggest the following: (1) f-circRNAs are involved in the mechanism of tumorigenesis, progression and therapy resistance; and (2) cell-free f-circRNAs could be a novel “liquid biopsy” biomarker to monitor the status of fusion genes in a noninvasive way. Therefore, we speculate and propose the following hypothesis: f-circRNAs of *the CLDN18-ARHGAP* fusion gene exist and can affect tumor function or act as a potential “liquid biopsy” biomarker for targeted drugs (e.g., zolbetuximab).

The present study also has some limitations. (1) This study only included five retrospective studies. Therefore, selection bias and quality deviation are likely among these studies, which may have an influence on the results of the meta-analysis. (2) The detection methods varied among the included studies. The potential false positive and false negative rates of *CLDN18-ARHGAP* fusion in the included studies may have influenced the results of the meta-analysis. (3) In addition, the limited follow-up duration of the included studies was another limitation of the presented studies. (4) Although previous studies successfully demonstrated that the *CLDN18-ARHGAP26* fusion gene can induce EMT, the loss of the epithelial phenotype, and cell-cell and cell-extracellular matrix adhesion, as well as increase the invasion ability and resistance to chemotherapy drugs in cancer cell lines (5, 6), the specific molecular mechanisms by which *CLDN18-ARHGAP26* regulates downstream molecules and pathways remain unclear. As different fusion modes generate various fusion proteins, it is difficult to design and develop antibodies to specifically target these fusion proteins, which may hinder mechanistic investigation of the *CLDN18-ARHGAP* fusion gene. Furthermore, large sample size multicenter studies are expected to validate the clinical significance and prognostic meaning of the *CLDN18-ARHGAP26* fusion gene in gastric cancer patients.

## Conclusions

*The CLDN18-ARHGAP* fusion gene is characterized as one of the features of diffuse gastric cancer. The *CLDN18-ARHGAP* fusion gene is correlated with advanced tumor stages in gastric cancer, as well as poor survival outcomes. Although *CLDN18-ARHGAP* fusion can increase the invasion and migration ability of gastric cancer cells *in vitro*, the molecular mechanism remains to be elucidated. Furthermore, the early detection of the *CLDN18-ARHGAP* fusion and targeted drugs for this fusion may potentially improve the survival outcomes of gastric cancer patients.

## Data Availability Statement

The raw data supporting the findings of this study are available from the corresponding author upon reasonable request.

## Author Contributions

W-HZ, HX, and J-KH designed the study. W-HZ, S-YZ, Q-QH, X-ZC, YQ, and YS collected information and analyzed and interpreted the data. Z-GZ, YS, HX, and J-KH supervised this study. All authors contributed to the article and approved the submitted version.

## Conflict of Interest

The authors declare that the research was conducted in the absence of any commercial or financial relationships that could be construed as a potential conflict of interest.

## Abbreviations

TCGA: The Cancer Genome Atlas
NOS: Newcastle–Ottawa Scale
SD: standard deviation
OR: odds ratio
MD: mean difference
HR: hazard ratio
CI: confidence intervals
GRAF: GTPase Regulator Associated with Focal Adhesion Kinase
EMT: Epithelial-Mesenchymal Transition
PDX: Patient-Derived Xenograft
RT-PCR: reverse transcription-polymerase chain reaction
FISH: Fluorescence *in situ* Hybridization.
